# The potential to improve Lyme disease diagnostics through quantification of immunoglobulin class switching patterns

**DOI:** 10.1128/jcm.01020-25

**Published:** 2025-09-26

**Authors:** Anna M. Schotthoefer

**Affiliations:** 1 Marshfield Clinic Research Institute, Marshfield Clinic Health System 5345 , Marshfield, Wisconsin, USA; Mayo Clinic Minnesota, Rochester, Minnesota, USA

## Abstract

N. Nair, A. Marques , E. J. Horn, G. Brown et al., J Clin Microbiol 63:e0034725, 2025, https://doi.org/10.1128/jcm.00347-25 present data to demonstrate that infection by *Borrelia burgdorferi*, the primary causative agent of Lyme disease in the USA, leads to immunoglobulin class switching in antibodies specifically against the immunodominant antigen, VlsE (variable major protein-like sequence, expressed), in a predictable pattern between the early acute (<1 month illness duration) to late-stage Lyme arthritis stages. Detection of anti-VlsE isotypes in sera was highly specific to Lyme disease patients, and the frequencies and abundances of IgM, IgG, and IgA isotypes progressed in a pattern consistent with the development of an anti-*B*. *burgdorferi* antibody response. Applying multivariate and machine learning modeling methods, they found the profile of isotypes quantified performed particularly well at correctly classifying early acute sera samples. They also identified IgG4 as a potential biomarker unique to Lyme arthritis patients. Overall, the findings suggest that improvements in Lyme disease diagnostics may be attained by quantifying specific antibody isotypes against *B. burgdorferi* antigens.

## COMMENTARY

Accurately assessing the public health burden of Lyme disease, the most commonly reported vector-borne disease in the USA, is challenging, in part, because of limitations in the currently available laboratory diagnostics, which are based on detecting the presence of antibodies against *Borrelia burgdorferi*, the primary etiologic agent of the disease in the USA. These serologic test methods provide indirect evidence of infection and are known to lack sensitivity in the acute stage of Lyme disease when antibodies have not yet developed to detectable levels in a significant proportion of patients (~40%–70%) (1, 2). Results of the tests also are often difficult to interpret when patients present with non-specific signs and symptoms, have a low pre-test probability, or have a prior history of infection (3, 4). Despite these challenges, the development of direct detection methods for *B. burgdorferi* is hindered by low concentrations and a fleeting presence of the pathogen in the blood of patients, such that efforts to improve serum antibody tests remain a major focus for developing better Lyme disease diagnostics (1, 5).

Current serodiagnosis methods for Lyme disease have depended on two-tier testing algorithms, the standard two-tier test (STTT) and the modified two-tier test (MTTT). These algorithms generally include detection of specific anti-*B*. *burgdorferi*, class-specific IgM and IgG immunoglobulin antibodies in the second-tier confirmatory tests (6, 7). IgM antibodies are important in the initial immune response to pathogens, often serving as the first antibodies to recognize the epitopes of invading organisms. Their detection is meant to be interpreted as signaling evidence of an acute Lyme disease infection. IgG antibodies, on the other hand, are produced during the secondary immune response, and they are viewed as signaling a later stage of Lyme disease; their detection in the absence of anti-*B*. *burgdorferi* IgM antibodies may suggest a past rather than active infection (1, 4, 8). However, there are limitations related to interpreting the results of these IgM and IgG tests because patients vary in their humoral immune responses and the presentation of Lyme disease signs and symptoms, which do not always follow a distinct linear, progressive pattern in association with the duration of the infection (9–11).

Having a diagnostic test that can reliably provide information about the stage of Lyme disease and particularly differentiate between acute and later stages of Lyme disease would help improve clinician treatment decisions, as the antibiotics and their duration recommended for treatment vary based on the stage of disease (2). Additionally, a test that might help distinguish between a likely active versus a resolved infection would be extremely valuable because once IgG antibodies are developed, they may persist for months to years. Not having the ability to distinguish between active and past infections using the current serologic-based diagnostics is particularly problematic for individuals living in highly endemic Lyme disease areas that may have a risk of being infected more than once over the course of their lifetime (12). Furthermore, an estimated 10%–20% of patients with Lyme disease go on to develop persisting symptoms after treatment in a condition known as post-treatment Lyme disease syndrome (PTLDS), while a subset of Lyme arthritis patients may suffer from antibiotic-refractory Lyme arthritis (2). The underlying causes of PTLDS and antibiotic-refractory Lyme arthritis are incompletely understood, but evidence is mounting to suggest that dysregulated proinflammatory immune responses or immune dysfunction involving autoimmunity are involved (e.g., references 13, 14).

Few studies have examined how specific antibodies change over time in infected individuals. Nair et al. (15) begin to address this knowledge gap by quantifying antibody isotypes against the *Borrelia*-specific VlsE (variable major protein-like sequence, expressed) protein in samples from patients at different stages of Lyme disease. VlsE is an immunodominant *Borrelia* protein and is an antigen used in current serologic diagnostic tests (1). Nair et al. found that the abundance of the major antibody classes measured in sera from patients with acute to late-stage Lyme disease progressed in sequence as might be predicted for development of an antibody B cell response (i.e., IgM→ IgG→ IgA). As such, IgM antibodies, which tend to be important during the primary B cell response, were most frequently detected and abundant (as measured by optical densities) in sera from early acute patients and declined in frequency and abundance in sera from early convalescent to late-stage Lyme arthritis patients. IgG class antibodies were frequently detected and abundant in sera from patients across all stages; however, Nair et al. demonstrated that the subclass, or isotype, of IgG antibodies varied in frequency and abundance with Lyme disease stage. IgG1 began with high frequency and abundance in sera from the early acute <1 month patients, peaked in abundance in the early acute >1 month patients, and then declined but remained high in the early convalescent and Lyme arthritis patient sera. IgG3 was high in both the early acute <1 month and >1 month patient sera but declined in the early convalescent sera. IgG4 was highest in the sera from Lyme arthritis patients and was detected at low frequency and abundance at the other stages. IgG2 was detected at low frequency and abundance at all stages. IgA1 was most abundant and frequently detected in sera from the early acute patients (both <1 month and >1 month).

Observing these differences, Nair et al. applied machine learning (ML) and multivariate analysis techniques, including single decision tree, random forests, and gradient boosted trees, to examine how well Lyme disease stages could be differentiated based on the levels of the various antibody isotypes in the samples. In general, detection of any anti-VlsE antibodies was highly predictive of Lyme disease, with 169 of 180 (94%) samples correctly classified as Lyme disease patients versus controls (Nair et al., Table 3; Table S1). The combined levels of isotypes measured in the study performed best at correctly predicting the early acute stages (<1 and >1 month), with about 70% of these samples correctly classified based on random forests (Nair et al., Table 3), and the ML methods, in general, demonstrated sensitivities and postive predictive values (PPVs) >50% and specificities and negative predictive values (NPVs) >90% (Nair et al., Table 2).

Although there were differences in the antibody isotype profiles observed in the Lyme arthritis and PTLDS sera tested by Nair et al. (e.g., Nair et al., Fig. 2), the multivariate and ML models performed poorly at correctly classifying these sera; for instance, only 17% and 16% of Lyme arthritis and PTLDS, respectively, were correctly classified by random forests (Nair et al., Table 3). Most of these samples were misclassified as early convalescent. Lyme arthritis, PTLDS, and early convalescent patient sera profiles were similar in displaying low levels of IgM and IgA1 isotypes and high levels of IgG1 and IgG3. Also of note was that 5 of 25 (20%) sera in the panel of PTLDS patients were misclassified as controls. Though this observation was based on a small sample size, few other sera from Lyme disease patients were misclassified as controls, suggesting it would be worth further exploring whether there are certain PTLDS patients that consistently will have low or undetectable anti-VlsE isotype antibodies in their sera, and if so, why this might be.

A potential unique feature Nair et al. found to be associated with Lyme arthritis sera was elevated IgG4 anti-VlsE antibodies. Further investigation is needed to determine if IgG4 might serve as a specific biomarker for Lyme arthritis and, importantly, if the pattern of isotype switching and induction of IgG4 is the result of *B. burgdorferi* infection and progression to the Lyme arthritis stage, or if elevation of the specific isotype reflects possible immune-driven pathogenesis associated with Lyme arthritis. Class switching to IgG4 generally can be associated with repeated exposure to an antigen and indicate antigen tolerance, as well as serve as a regulator of inflammation (16). As such, its detection in Lyme arthritis patients may reflect class switching induced by the inflammatory processes associated with the repeated exposure to *B. burgdorferi* antigens that may occur over the typically longer course of infections in Lyme arthritis patients. Once antibiotic-responsive Lyme arthritis patients receive treatment, it is expected that the inflammatory processes will be downregulated, and therefore, elevated levels of IgG4 may signal a controlled, or possibly even recovered, infection (17, 18). In contrast, IgG4 antibodies are known to also be associated with pathogenic responses and specifically antibody-mediated autoimmunity (16). Because there is evidence that antibiotic-refractory Lyme arthritis is associated with autoantibody-dependent pathogenesis (17, 18), to distinguish between these two possibilities, and to understand how the elevated IgG4 antibodies detected in Lyme arthritis sera should be interpreted, it would be necessary to know if IgG4 levels vary between Lyme arthritis patients that respond to versus those that are refractory to antibiotic treatment.

Another potential shortfall of the anti-VlsE antibody isotyping profiling method that needs further evaluation is how well it might perform in patients who have not yet developed IgM and IgG antibodies detectable by STTT or MTTT. All sera in the Lyme disease stage panels used in the study had detectable antibodies by STTT, whereas blinded sera from a collection of acute seronegative patients and healthy controls displayed little anti-VlsE seroreactivity. It is not clear from Nair et al.’s results how well the results in the blinded samples aligned with the health status of the donors as seronegative Lyme disease patients versus controls, or if there were any anti-VlsE antibodies detected in sera from patients that were seronegative by STTT. Knowing whether there are anti-VlsE isotype antibodies that are detectable in patients that are seronegative by STTT or MTTT would be important for understanding if improvements in the sensitivity of Lyme diagnostics could be made by designing assays specific to such early isotypes. For instance, the detection of IgG1, IgG3, and IgA1 isotypes in early acute <1 month patient sera in the study suggests that detection of such specific isotypes may provide improvements in sensitivity.

The authors did not discuss their findings in relation to the clinical details about the patients included in the sera panels they examined in the study, as they may not have had access to this information; however, it is possible that future improvements in an ML classifier may be attained by including more specific clinical variables in the models, such as whether one or more erythema migrans lesions were present, the duration of signs and symptoms, as well as length of antibiotic treatment at the time of sera collection. Such input features would likely help reveal clinical features specifically associated with optimizing correct ML model classifications and identify the potential applications that antibody isotyping and profiling, in general, may offer for improving Lyme diagnostics.

In summary, an ideal Lyme disease diagnostic test would (i) have improved sensitivity, without compromised specificity, in acute stage Lyme disease, (ii) be able to help discriminate stages of Lyme disease, and (iii) be able to distinguish between active and resolved infections. The results presented by Nair et al. support the hypothesis that these goals may be achieved by integrating information about the quantities of specific antibody classes and isotypes present in patient sera. Future studies would benefit from comparisons of isotype profiles in serial collections of sera from patients presenting with evidence of acute versus late-stage Lyme disease, beginning with collection at the time of clinical presentation through to several post-treatment time points. Such analyses would provide the necessary insight into how best to integrate information about these isotype profiles into improved serodiagnosis for Lyme disease.
